# A novel inhibitor of *Chlamydophila pneumoniae *protein kinase D (PknD) inhibits phosphorylation of CdsD and suppresses bacterial replication

**DOI:** 10.1186/1471-2180-9-218

**Published:** 2009-10-14

**Authors:** Dustin L Johnson, Chris B Stone, David C Bulir, Brian K Coombes, James B Mahony

**Affiliations:** 1 MG DeGroote Institute for Infectious Disease Research and the Department of Pathology and Molecular Medicine, McMaster University, Ontario, Canada; 2 The Father Sean O'Sullivan Research Centre, St Joseph's Healthcare, Hamilton, Canada

## Abstract

**Background:**

We have shown previously that *Chlamydophila pneumoniae *contains a dual-specific Ser/Thr protein kinase that phosphorylates CdsD, a structural component of the type III secretion apparatus. To further study the role of PknD in growth and development we sought to identify a PknD inhibitor to determine whether PknD activity is required for replication.

**Results:**

Using an *in vitro *kinase assay we screened 80 known eukaryotic protein kinase inhibitors for activity against PknD and identified a 3'-pyridyl oxindole compound that inhibited PknD autophosphorylation and phosphorylation of CdsD. The PknD inhibitor significantly retarded the growth rate of *C. pneumoniae *as evidenced by the presence of very small inclusions with a reduced number of bacteria as seen by electron microscopy. These inclusions contained the normal replicative forms including elementary bodies (EB), intermediate bodies (IB) and reticulate bodies (RB), but lacked persistent bodies (PB), indicating that induction of persistence was not the cause of reduced chlamydial growth. Blind passage of *C. pneumoniae *grown in the presence of this PknD inhibitor for 72 or 84 hr failed to produce inclusions, suggesting this compound blocks an essential step in the production of infectious chlamydial EB. The compound was not toxic to HeLa cells, did not block activation of the MEK/ERK pathway required for chlamydial invasion and did not block intracellular replication of either *Chlamydia trachomatis *serovar D or *Salmonella enterica *sv. Typhimurium suggesting that the inhibitory effect of the compound is specific for *C. pneumoniae*.

**Conclusion:**

We have identified a 3'-pyridyl oxindole compound that inhibits the *in vitro *kinase activity of *C. pneumoniae *PknD and inhibits the growth and production of infectious *C. pneumoniae *progeny in HeLa cells. Together, these results suggest that PknD may play a key role in the developmental cycle of *C. pneumoniae*.

## Background

*Chlamydophila pneumoniae *is an important human respiratory pathogen that causes laryngitis, pharyngitis, bronchitis and community acquired pneumonia [1] and has been associated with exacerbation of asthma [2,3], atherosclerosis [4-6], arthritis [2,7], Alzheimer's disease [8,9] and Multiple Sclerosis [10-13]. The ability of *C. pneumoniae *to remain viable within lung macrophages [14-16] provides a mechanism for dissemination of *Chlamydia *to other anatomical sites that may include the arterial wall [17] and the brain. Rapid and successful treatment of *C. pneumoniae *respiratory infections is therefore important to ensure complete clearance of the bacteria in order to avoid infections elsewhere in the body. Antibiotics such as azithromycin, clarithromycin, erythromycin, and doxycycline have been used to treat *C. pneumoniae *respiratory infections [18]. However, clinical isolates of *Chlamydia *resistant to azithromycin and erythromycin have been reported [19], and some chlamydial species including *C. pneumoniae *develop resistance to antibiotics *in vitro *[20-25]. Furthermore, sub-optimal concentrations of antibiotics *in vivo *may result in chlamydial persistence [16,26], rendering the bacteria refractory to further antibiotic therapy [27,28], and increasing the likelihood of *Chlamydia *persisting in the body for months or years [29,30]. Given that persistent chlamydial infections may lead to chronic conditions there is a need to develop novel anti-microbials to eradicate chlamydial infections.

All chlamydiae *spp*. exhibit a developmental cycle that begins when an infectious elementary body attaches to and invades a eukaryotic host cell. During invasion the EB becomes enveloped by the host cell plasma membrane, ultimately creating an intracellular vacuole known as an inclusion, within which the bacterium undergoes replication. The EB next transforms into a reticulate body, a developmental process that is characterized by reduction of EB outer membrane proteins [31-33] and DNA decondensation. RB are non-infectious, 2-5 times larger than EB and metabolically active. Division of RB occurs once every 2-3 hours for *C. trachomatis *and 6-7 hours for *C. pneumoniae *[34-36]. A hallmark of chlamydial replication is the expansion of the host cell-derived inclusion membrane to accommodate increasing numbers of bacteria. In response to an as yet unidentified signal, RB begin to asynchronously differentiate into infectious EB by transformation through the IB stage that contains partially condensed chromosomal DNA. The end of the developmental cycle occurs when EB are released from the host cell following inclusion lysis, or extrusion of the inclusion into neighbouring cells [37]. In addition to the three developmental forms seen during the chlamydial developmental cycle, *Chlamydia *may be induced to form persistent bodies, a morphological state not part of normal growth and development. The PB is an abnormally large form of chlamydia that occurs in response to interferon-γ [27], antibiotics [26], or iron limitation [38], and is characterized by an inability to segregate into daughter cells after genomic DNA replication. The arrest of the developmental cycle at the PB stage can be reversed when the inducer stimulus in the case of iron deprivation is removed [38]. In addition to interferon-γ, and conventional antibiotics such as β-lactams and macrolides, other compounds exhibit bacteriostatic activity against *Chlamydia *in cell culture. These include selective cycloxygenase inhibitors, rottlerin and inhibitors of type III secretion [34,38-42]. Rottlerin is a pan-specific inhibitor of eukaryotic protein kinases and was recently shown to inhibit the growth of *C. pneumoniae *in HeLa cells [40]. Rottlerin may interfere with activation of the host MEK/ERK pathway which has been shown to be necessary for chlamydial cell invasion [43] and therefore indirectly cause inhibition of chlamydial growth. Alternatively, INP0007 (compound C1), INP0010, and INP0400, inhibitors of *Yersinia *type III secretion, may target a bacterial-specific factor related to the type III secretion system and directly abrogate chlamydial growth in eukaryotic cells [39,41,44]. The identification of novel targets may prove useful in the development of new antimicrobials effective against chlamydiae.

Chlamydial genomic studies have identified three Ser/Thr protein kinases, Pkn1, Pkn5, and PknD. Our laboratory has shown previously that *C. pneumoniae *PknD is a dual-specific protein kinase that autophosphorylates on threonine and tyrosine residues and phosphorylates serine and tyrosine residues of the FHA-2 domain of Cpn0712, a putative Yersinia YscD ortholog called CdsD [45]. In this report we show that a 3'-pyridyl oxindole compound, a known inhibitor of Janus kinase 3 (JAK3), inhibits *C. pneumoniae *PknD activity. This compound prevented PknD autophosphorylation and phosphorylation of CdsD, a type III secretion apparatus protein. When added to infected HeLa cells, the compound retarded *C. pneumoniae *growth and significantly reduced the amount of infectious *C. pneumoniae *produced suggesting that PknD plays an important role in chlamydial replication.

## Results

### Identification of an inhibitor of *C. pneumoniae *PknD protein kinase activity

**Figure 1:**
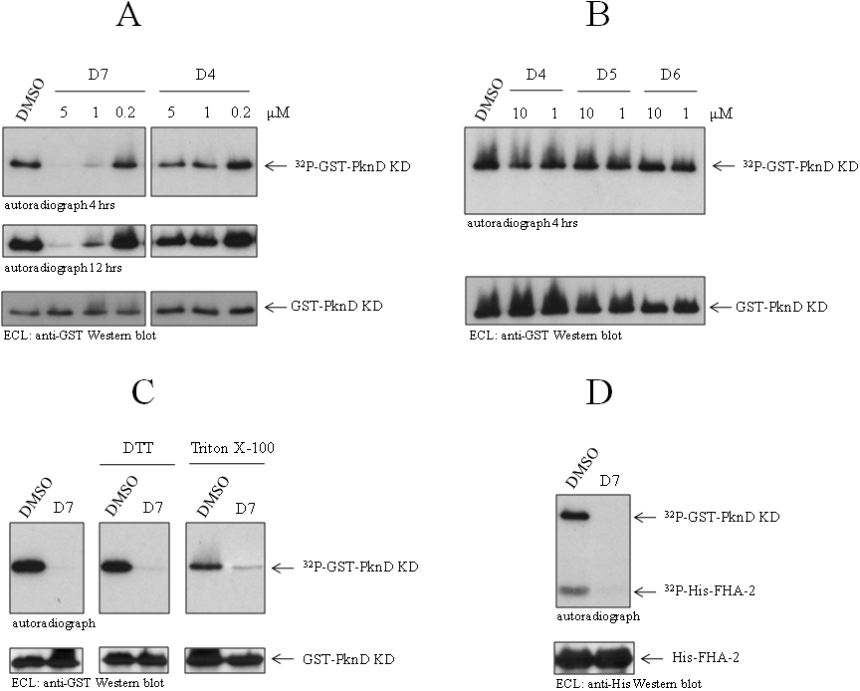
**Inhibition of PknD by compound D7**. A: compound D7, but not compound D4 or DMSO, substantially inhibited PknD autophosphorylation (^32^P-GST-PknD KD) at 5 and 1 μM, as seen by autoradiography. B: related compounds D5 and D6 did not inhibit PknD at 1 or 10 μM. C: 1 mM DTT and 1% Triton X-100 did not decrease inhibition of PknD by compound D7 (used at 10 μM in all panels). DMSO (0.1%) is shown as control. D: compound D7 inhibited phosphorylation of the FHA-2 domain (^32^P-His-FHA-2) of CdsD by PknD. Western blotting showed equivalent amounts of protein in each autoradiograph (lower panels).

We have recently shown that *C. pneumoniae *contains three Ser/Thr protein kinases [46] and that one of these, PknD, phosphorylates CdsD, a structural component of the type III secretion system (T3SS) [45]. In order to determine whether PknD plays an essential role in *Chlamydia *development, we screened an existing library of 80 small molecule kinase inhibitors, including inhibitors of eukaryotic receptor tyrosine kinases and atypical kinases, for their ability to inhibit PknD autophosphorylation *in vitro*. Recombinant GST-tagged PknD kinase domain (GST-PknD KD) was pre-incubated with 10 μM of each compound and reactions initiated with the addition of kinase assay buffer containing Mn^2+ ^and ATP. SDS-PAGE and Western blotting followed by autoradiography was used to visualize the extent of PknD autophosphorylation in the presence of each compound. Nine compounds (EMD designations: D7, E8, F4, F5, F6, F7, G5, H10, and H11) of the 80 tested exhibited some level of inhibition of PknD autophosphorylation when tested at 10 μM (data not shown). Of these nine compounds only one, compound D7, a 3'-pyridyl oxindole, completely inhibited PknD autophosphorylation. Fig. 1A shows a dose response for PknD inhibition. At 1 μM compound D7 reduced PknD autophosphorylation by greater than 50% (fig. 1A). Similar results were obtained with two different lots of the inhibitor. Compound D4, a pan-specific inhibitor of the Janus kinase (JAK) family, did not significantly inhibit PknD autophosphorylation at concentrations of 0.2 to 10 μM (figs. 1A and 1B). Similarly, two other JAK3 inhibitors, compounds D5 and D6, did not inhibit PknD autophosphorylation at concentrations of 1 or 10 μM (fig. 1B).

Compound D7 is ATP competitive and therefore it has the potential to inhibit other chlamydial enzymes that utilize ATP as a substrate. To determine if compound D7 could inhibit a chlamydial ATPase, we examined its effect on the activity of CdsN, the T3SS ATPase of *C. pneumoniae *[47]. The activity of CdsN was 0.51 ± 0.09 and 0.43 ± 0.06 micromoles of phosphate/min/mg protein in the presence of 5 μM and 100 μM of compound D7, respectively, compared with 0.46 ± 0.04 in the absence of compound D7. Compound D7 did not inhibit CdsN activity suggesting that it may not be a broad spectrum inhibitor of enzymes that utilize ATP as a substrate.

To assess whether compound D7 could be used in cell culture we first exposed the compound to reducing conditions similar to that found in eukaryotic cells, then tested its ability to inhibit PknD. Equivalent volumes of compound D7 (100 μM) and DTT (2 mM) were mixed on ice for 15 minutes prior to testing in the kinase assay. Compound D7 retained the ability to inhibit PknD autophosphorylation (fig. 1C) after exposure to DTT, suggesting that it would not have decreased effectiveness under the reducing conditions of the cell cytoplasm. To rule out the possibility that the inhibitory effect of D7 was due to aggregates of the compound, we tested for inhibitory activity in the presence of 1% Triton X-100 to reduce potential aggregates. Compound D7 retained efficacy toward PknD in the presence of 1% Triton X-100 (fig. 1C), indicating that the inhibition was not due to a non-specific effect of compound D7 aggregates.

We recently identified CdsD, an ortholog of *Yersinia *YscD, as a substrate of PknD and showed that PknD phosphorylated 2 FHA domains of CdsD [45]. We therefore examined whether compound D7 could block phosphorylation of CdsD by PknD. Compound D7 completely blocked the phosphorylation of the CdsD FHA-2 domain by PknD (fig. 1D) indicating that, in addition to inhibiting PknD autophosphorylation, it also inhibits phosphorylation of CdsD.

### Effect of compound D7 on the growth of *C. pneumoniae *in HeLa cells

**Figure 2:**
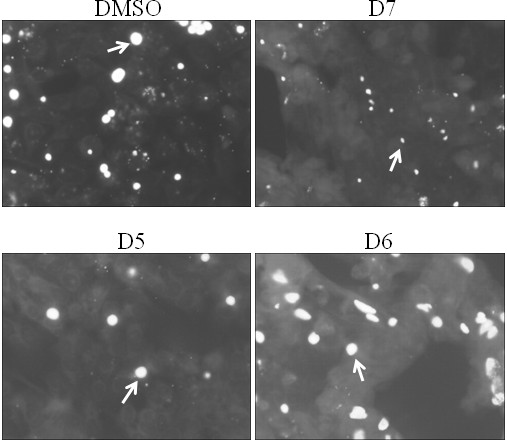
**Compound D7 inhibits the growth of *C. pneumoniae *in HeLa cells**. Detection of inclusions at 72 hpi by IF microscopy revealed very small inclusions when *C. pneumoniae*-infected HeLa cells were exposed to 10 μM of compound D7, but not when exposed to DMSO (0.1%) or 10 μM of compounds D5 or D6. Arrows indicate representative inclusions. Inclusions were stained with FITC-conjugated anti-LPS monoclonal antibody containing Evan's Blue counterstain.

The identification of a PknD inhibitor provides a new tool to study the role of PknD in the developmental cycle of *C. pneumoniae*. Since PknD may play a role at various times throughout the 72 hour developmental cycle we tested the effect of several compounds including compound D7 on the growth of *C. pneumoniae *in cell culture. Compounds were added to the cell culture media 1 hr prior to infection with *C. pneumoniae *and inclusions were visualized by immunofluorescent (IF) staining at 72 hr. Compound D7 retarded the growth of *C. pneumoniae *in HeLa cells (fig. 2) as indicated by the presence of very small inclusions at 72 h. Compounds D5, D6 and vehicle (0.1% DMSO) did not have any effect on the development of inclusions judged by the presence of normal size inclusions. Given that compounds D5, D6 and D7 are JAK3 kinase inhibitors, and only compound D7 affects growth of *C. pneumoniae*, JAK3 inhibition is not likely responsible for the decreased chlamydial growth rate.

### Compound D7 exhibits a dose-dependent but time-independent effect on *C. pneumoniae *growth

**Figure 3:**
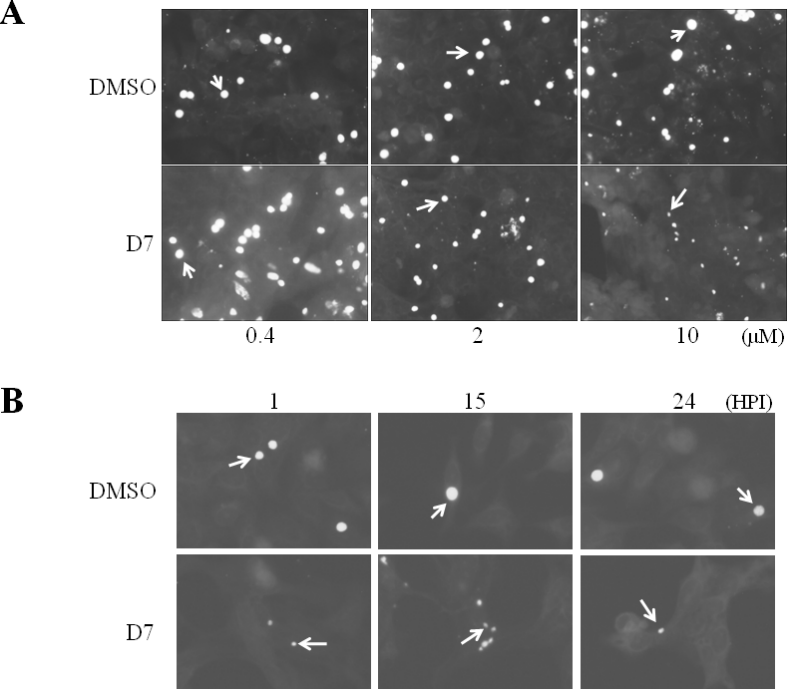
***C. pneumoniae *growth inhibition by compound D7 is dose-dependent**. A: compound D7 at 0.4 μM exhibited no inhibition of chlamydial growth (normal size inclusions), 2 μM exhibited partial inhibition (smaller inclusions), and 10 μM had a significant inhibitory effect (significantly reduced inclusion size) (bottom panels, left to right, respectively). DMSO controls at 0.004, 0.02, and 0.1% (top panels, left to right, respectively) did not restrict growth as indicated by inclusion size. Arrows indicate representative inclusions. B: Addition of 10 μM compound D7 to *C. pneumoniae*-infected HeLa cells at 1, 15 or 24 hpi resulted in small inclusions at 72 hpi. Inclusions were stained with FITC-conjugated anti-LPS monoclonal antibody containing Evan's Blue counterstain.

To determine whether the effect of compound D7 on chlamydial growth is dose-dependent we added compound D7 to infected HeLa cells at 1 hr post infection at final concentrations of 0.4, 2 and 10 μM and assessed inclusion size at 72 hpi. Vehicle or 0.4 μM of D7 resulted in normal size inclusions at 72 hr (fig. 3A). Compound D7 at 2 μM resulted in slightly smaller inclusions relative to DMSO-only exposure while D7 at 10 μM resulted in very small inclusions (fig. 3A). To determine if compound D7 exerts a time-dependent effect on *Chlamydia *growth, the compound was added to infected cells at 15 and 24 hours post infection in addition to 1 hpi. Under each condition inclusions were very small at 72 hpi compared to inclusions in cells exposed to vehicle (fig. 3B) indicating that the effect of compound D7 on *Chlamydia *growth is not restricted to a time prior to 24 hpi. These results demonstrate that compound D7 exerts a dose-dependent but time-independent effect on *C. pneumoniae *growth in HeLa cells.

### Compound D7 does not affect HeLa cell viability

**Figure 4:**
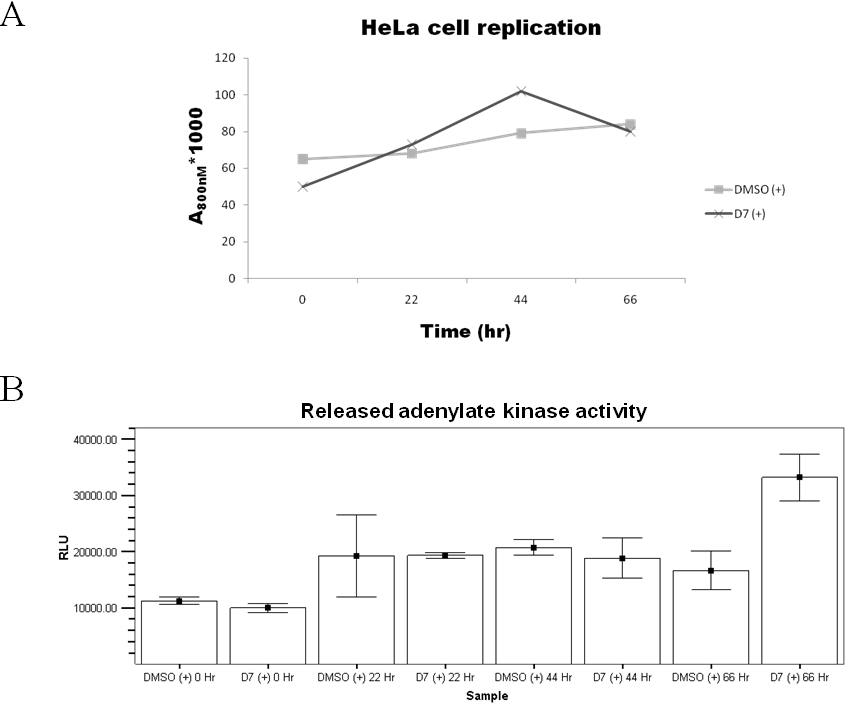
**Compound D7 does not reduce HeLa cell viability**. A: subconfluent HeLa cell monolayers incubated in MEM containing either DMSO (0.1%) or compound D7 (10 μM) with 2 μg/mL cycloheximide (+), were collected by trypsinization and the cell density was measured by absorbance at 800 nM at the times indicated. Compound D7 did not significantly alter HeLa cell number compared to DMSO alone. B: cell culture supernatant adenylate kinase activity from the samples in (A). Exposure of HeLa cells to 10 μM compound D7 for 44 hours was not more cytotoxic than cells exposed to DMSO. At 66 hours there was a small increase in HeLa cell release of adenylate kinase in the D7-exposed group. Error bars represent means plus 2 standard deviations.

Since inhibition of *C. pneumoniae *growth could be due to an effect of compound D7 on host cell viability, we assessed whether D7 affects HeLa cell replication and cytotoxicity. Uninfected HeLa cells were incubated in the presence of 10 μM compound D7 or DMSO, and cell density was assessed at 0, 22, 44 and 66 hours using a spectrophotometric assay. Compound D7 had little or no effect on HeLa cell growth rate compared to DMSO (fig. 4A). We also examined cell cytotoxicity at these times using an adenylate kinase release assay. Compound D7 exhibited the same level of cytotoxicity as DMSO at 0, 22 and 44 hours, and only slightly higher cytotoxicity levels at 66 hr compared to DMSO-exposed cells (fig. 4B). Therefore compound D7 had little or no effect on HeLa cell viability and the inhibitory effect of D7 on chlamydial growth is not likely due to a non-specific cytotoxic effect on the host cell.

### Compound D7 does not block activation of the MEK/ERK pathway

**Figure 5:**
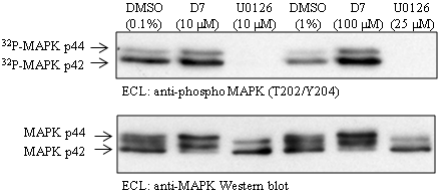
**Compound D7 does not block activation of the MEK/ERK pathway in EGF-stimulated HeLa cells**. HeLa cells incubated with DMSO, compound D7 or U0126 were activated with EGF and the levels of MAP kinase phosphorylation were determined by Western blot using anti-phospho ERK1/2 antibody. Compound D7 at 10 and 100 μM, and DMSO at 0.1 and 1%, did not prevent phosphorylation of MAP kinase following EGF stimulation of HeLa cells. U0126 at 10 and 25 μM completely prevented phosphorylation of MAP kinase. Blots were probed with antibody to phosphorylated MAPK (upper panel), and with antibody to total MAPK (lower panel).

It has been shown previously that activation of the MEK/ERK pathway is necessary for chlamydial invasion of host cells [43] and sustained activation of this pathway is required for acquisition of host glycerophospholipids by *Chlamydia *[48]. To rule out the possibility that the inhibitory effect of compound D7 on *C. pneumoniae *growth could be due to an inhibition of the MEK/ERK pathway we assessed the level of ERK1 and ERK2 (p44/p42 MAP kinase, respectively) phosphorylation in the presence of compound D7. HeLa cells exposed to either 10 or 100 μM of compound D7 contained high levels of phosphorylated p44 and p42 MAP kinase following EGF stimulation. HeLa cells exposed to 10 or 25 μM U0126, a specific inhibitor of MEK1/2, were used as control and did not contain phosphorylated p44 or p42 MAP kinase following EGF stimulation (fig. 5). This result demonstrates that compound D7 does not block phosphorylation of p44/p42 MAP kinase in HeLa cells, suggesting that chlamydial growth inhibition caused by D7 was not due to a non-specific blockage of the MEK/ERK pathway.

### Effect of compound D7 on the growth of *Salmonella enterica *sv. Typhimurium and *C. trachomatis *serovar D

**Figure 6:**
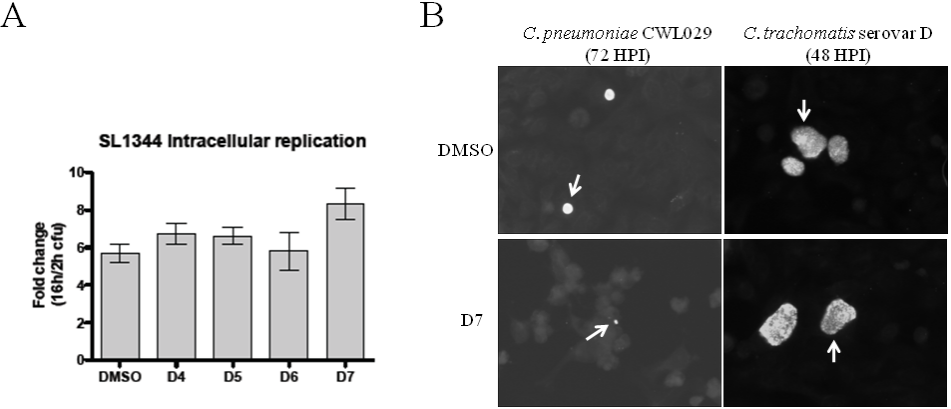
**Compound D7 does not inhibit the growth of *Salmonella enterica *sv. Typhimurium or *C. trachomatis *serovar D in HeLa cells.** A: compounds D4, D5, D6 and D7 (10 μM) or DMSO (0.1%), did not prevent replication of *Salmonella enterica *sv. Typhimurium SL1344 in HeLa cells. Compounds were added to the media 2 hours after host cell infection, and bacteria harvested at both 2 and 16 hpi in order to plot the fold change in colony forming units. B: compound D7 did not inhibit the growth of *Chlamydia trachomatis *serovar D. Compound D7 (10 μM) was added to cell monolayers 1 hpi and inclusions were stained at 48 hpi. Large inclusions were seen in both D7- (bottom right panel) and DMSO-exposed (0.1%; top right panel) cells while small inclusions were seen for *C. pneumoniae *in D7-exposed cells. Arrows indicate representative inclusions. The monoclonal antibody contained Evan's Blue counterstain for detection of host cells.

Since compound D7 could inhibit *C. pneumoniae *growth indirectly by affecting a common signaling pathway of the host cell, we examined the effect of compound D7 on the growth of another intracellular bacterial pathogen, *Salmonella enterica *sv. Typhimurium SL1344. Compound D7, as well as compounds D4, D5, D6 and DMSO, did not inhibit *Salmonella *replication in HeLa cells (fig. 6A), suggesting that the inhibitory effect of D7 was specific to *C. pneumoniae *and not the result of interference with a common signaling pathway of the host cell related to intracellular pathogens. To determine whether compound D7 was inhibiting a host signaling pathway or cellular function used by the chlamydiae *spp*. we examined the growth of *Chlamydia trachomatis *serovar D in HeLa cells in the presence of compound D7. Compound D7 did not inhibit the growth of *C. trachomatis *in HeLa cells as assessed by IF staining of mature inclusions present at 48 hr (fig. 6B), indicating that compound D7 is specific for *C. pneumoniae*, does not inhibit *C. trachomatis*, and does not block a common signaling pathway used by chlamydiae *spp*.

### Compound D7 does not cause chlamydial persistence and does not block differentiation or replication

**Figure 7:**
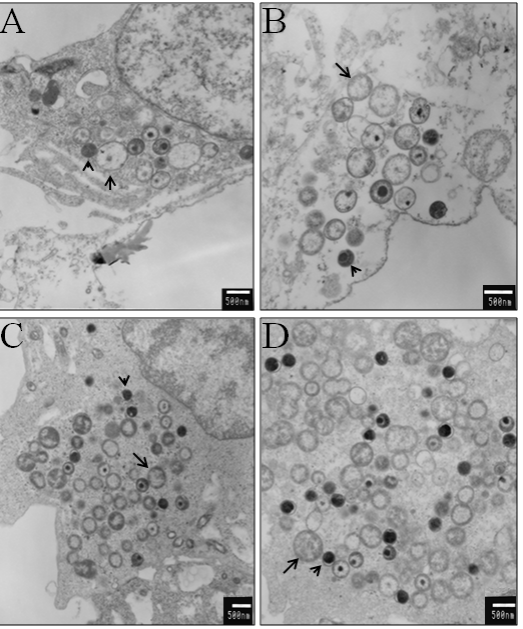
**Normal developmental forms of *C. pneumoniae *are found within compound D7-exposed inclusions**. At 48 hpi, infected HeLa cells incubated in MEM containing 10 μM of either compound D6 or D7 were observed by TEM. A, B: inclusions in D7-exposed cells are smaller and contain fewer bacteria, but all three developmental forms (EB, IB and RB) of *C. pneumoniae *are present. C, D: *C. pneumoniae *inclusions exposed to compound D6 are normal in size and contain the same normal developmental forms. Size bars are indicated in white (500 nm). Representative micrographs indicating RB (arrows) and EB (arrow heads) are shown.

Since the evidence indicates the inhibitory effect of compound D7 on *Chlamydia *growth can be exerted early in the developmental cycle (between 1-24 hpi), it is possible that the inhibitory effect occurs at a specific stage *viz*. EB to RB differentiation or RB replication. Alternatively, a block in replication could be due to the induction of persistence which occurs under conditions of limiting tryptophan or iron. To determine whether compound D7 blocks chlamydial growth at a specific stage, we used electron microscopy to look for various developmental forms. Figs. 7A and 7B show representative inclusions at 48 hpi from *C. pneumoniae*-infected HeLa cells incubated in the presence of 10 μM compound D7. These inclusions are smaller and contain fewer bacteria compared with chlamydial inclusions in the absence of compound D7 (figs. 7C and 7D), consistent with results seen using IF staining. All three developmental forms of *Chlamydia*, (EB, IB and RB) were seen in the presence of compound D7, and no aberrant forms or PB were detected, indicating that the inhibition of chlamydial growth was not due to the induction of persistent bodies. These results show that compound D7 attenuates *Chlamydia *growth by decreasing the number of bacteria present in infected cells.

### Compound D7 decreases the number and infectivity of *C. pneumoniae *progeny

**Figure 8:**
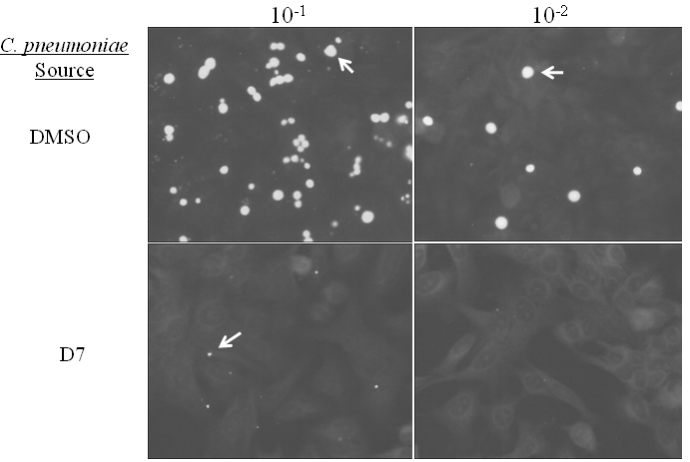
**Compound D7 reduces the number and infectivity of *C. pneumoniae *progeny**. HeLa cells were infected with *C. pneumoniae *(MOI of 5) and MEM containing either DMSO (0.1%) or D7 (10 μM) was added at 1 hpi. Cells were lysed at 72 hpi and chlamydial lysates diluted 10^-1 ^and 10^-2 ^and used to infect fresh HeLa cell monolayers. Infected cells were then incubated for 72 hours in MEM (without D7 or DMSO) and inclusions were stained with FITC-conjugated anti-LPS monoclonal antibody. *C. pneumoniae *harvested from DMSO-exposed HeLa cells produced many inclusions of normal size upon subsequent passage (top panels; dilution of passage indicated). A substantial reduction in both the number and size of inclusions was seen with chlamydiae harvested from HeLa cells exposed to compound D7 (bottom panels). Similar results were obtained with undiluted chlamydial lysates and with lysates harvested at 84 hpi (data not shown).

To determine whether *Chlamydia *progeny are infectious after exposure to compound D7, a blind passage experiment was performed. *C. pneumoniae*-infected HeLa cells were incubated in the presence of compound D7 or DMSO and the cells were lysed at 72 or 84 hr. Lysates containing chlamydiae were either undiluted, or diluted in media lacking compound D7 and blind passaged onto fresh HeLa cell monolayers. Compound D7 reduced the number of infectious chlamydiae compared with DMSO alone at both times by greater than 90% based on inclusion counts (fig. 8). In addition to reducing the number of inclusions, compound D7-exposed *C. pneumoniae *produced inclusions that were smaller in size compared to unexposed cultures, consistent with results seen on first passage (figs. 2, 3). These results indicate that compound D7 decreases the number and infectivity of *C. pneumoniae *progeny.

## Discussion

Chlamydiae are obligate intracellular pathogens that have a unique biphasic developmental cycle. We have previously shown that *C. pneumoniae *contains three Ser/Thr protein kinases and that one of these, PknD, is a membrane-associated kinase that phosphorylates CdsD, a structural protein of the type III secretion system [45]. In the present study we have identified a selective inhibitor of PknD and show that this compound blocks phosphorylation of CdsD *in vitro*, retards the intracellular growth rate and decreases the number of infectious *C. pneumoniae *produced following infection of HeLa cells.

To elucidate the role of PknD in the chlamydial developmental cycle, we screened a small library of known eukaryotic kinase inhibitors in an attempt to identify a PknD inhibitor. In this study we show that compound D7 is a potent inhibitor of *C. pneumoniae *PknD activity *in vitro*. PknD autophosphorylation and subsequent phosphorylation of the substrate CdsD were completely inhibited by compound D7. When added to *C. pneumoniae*-infected HeLa cells, the 3' pyridyl oxindole compound retarded chlamydial replication. The restriction of the developmental cycle was not due to the induction of chlamydial persistence as seen with interferon-γ or iron deprivation [34,38] since PB were not detected in inclusions when viewed by electron microscopy. Compound D7 also decreased the number of infectious *C. pneumoniae *upon passage suggesting that the compound interferes with an essential step in *C. pneumoniae *development.

The mechanism of chlamydial growth retardation by compound D7 is unknown but an involvement of host cell JAK3 is unlikely because the expression of JAK3 is restricted to the hematopoietic cell lineage [49-51] and HeLa cells do not express JAK3. The absence of JAK3 in *Chlamydia*-infected HeLa cells is supported by a recent study that failed to detect the induction or expression of the JAK3 substrate, STAT5, in *C. trachomatis*-infected HeLa cells [52]. In addition, other potent JAK3 inhibitors (compounds D4, D5 and D6) did not interfere with *C. pneumoniae *growth in HeLa cells. Therefore the mechanism of *C. pneumoniae *growth retardation in HeLa cells is unlikely due to an effect of compound D7 on JAK3 activity.

Our data also rule out an effect of compound D7 on the MEK/ERK signaling pathway required for chlamydial infection and intracellular growth. Activation of the MEK/ERK pathway has been shown to be essential for chlamydial invasion of HeLa cells [43], and sustained activation of Raf-MEK-ERK-cPLA2 is also required for acquisition of glycerophospholipids and growth by *C. pneumoniae *[48]. In our experiments 100 μM of compound D7 (10 fold higher than the concentration that inhibits chlamydial replication) did not interfere with MAP kinase phosphorylation in response to EGF, indicating that compound D7 does not block activation of the MEK/ERK pathway and that interference with this signaling pathway is not the mechanism of compound D7-mediated growth retardation.

Since protein kinase inhibitors are known to be promiscuous [53-55] and compound D7 could inhibit a kinase or other enzyme required for the growth of *C. pneumoniae*, a similar growth inhibition by compound D7 might be expected for other intracellular bacteria. Since compound D7 did not inhibit the growth of *Chlamydia trachomatis *serovar D or *Salmonella enterica *sv. Typhimurium SL1344, an effect of D7 on a common signaling pathway used by intracellular pathogens is not likely the mechanism of *C. pneumoniae *growth retardation.

Our results show that compound D7 inhibits the autophosphorylation of PknD and subsequent phosphorylation of *C. pneumoniae *CdsD *in vitro *and significantly retards the growth of *C. pneumoniae *in HeLa cells. However, our data does not allow us to state unequivocally that the reduced rate of growth in the presence of compound D7 is directly due to inhibition of PknD activity. Our attempts to detect phosphorylated CdsD *in vivo *by mass spectrometry have not been successful as it is technically difficult to harvest enough CdsD protein suitable for this method. We are exploring other methods for detecting CdsD phosphorylation *in vivo *as the detection of the phosphorylation status of PknD or CdsD in the presence of compound D7 would allow us to make a stronger link between PknD activity and growth rate. Since *C. trachomatis *contains a PknD ortholog we might expect compound D7 to affect *C. trachomatis *but this is not the case as compound D7 did not affect the growth of *C. trachomatis *in HeLa cells. However, the limited homology between the catalytic domains of the PknD orthologs in *C. trachomatis *and *C. pneumoniae *might explain the differential effect of compound D7 on their respective growth rates. We are presently initiating experiments to assess whether compound D7 has any inhibitory effect on PknD orthologs of other chlamydial species and to determine effects on bacterial replication rates.

Electron microscopic examination of *Chlamydia*-infected cells exposed to compound D7 revealed the presence of very small inclusions with significantly reduced numbers of bacteria. Inclusions contained all 3 developmental forms including RB, EB and IB and therefore both replication and differentiation of *C. pneumoniae *occurred in the presence of D7, albeit at a reduced rate. If inhibition of PknD is the mechanism by which compound D7 exerts its inhibitory effect on chlamydial replication, the presence of replicating RB in inclusions indicates that PknD activity is not essential for bacterial replication. In this scenario one could envisage a redundant or compensatory signaling pathway that circumvents the effect of compound D7-mediated PknD inhibition. Alternatively, PknD may be involved in a signaling pathway indirectly related to replication and that when inhibited only slows the rate of replication. It is also possible that PknD is an essential enzyme required for replication, but is only partially inhibited in cell culture by the concentration of compound D7 used in our growth experiments. Indeed, it is known that chlamydial isolates can be heterogeneous in nature and therefore a subpopulation of *Chlamydia *may have been partially resistant to the effects of compound D7. Nonetheless, *C. pneumoniae *grown in the presence of compound D7 and subsequently passaged onto fresh HeLa cell monolayers failed to propagate and develop inclusions suggesting PknD may also be involved in the production of infectious bacteria. Inhibition of PknD could manifest as multiple biological effects if there is more than one PknD substrate, or if the affected biological events are linked. More work is needed to elucidate the role of PknD and the exact mechanism by which compound D7 inhibits the growth and development of *C. pneumoniae*. These experiments, however, will be difficult to conduct in the absence of a genetic transformation system for chlamydiae.

## Conclusion

We have identified a novel inhibitor of *C. pneumoniae *growth and development, and its biological effects may be mediated via inhibition of PknD. It is tempting to speculate that PknD plays an essential role in the developmental cycle of *C. pneumoniae*, which may include a role in replication and/or in the production of infectious progeny, but this hypothesis cannot be directly tested in the absence of a PknD knockout. The approach of using novel chemicals in cell culture to inhibit other Ser/Thr protein kinases of chlamydiae *viz*. Pkn1 or Pkn5 may prove fruitful in elucidating their roles in chlamydial development.

## Methods

### Reagents and Cell Lines

Minimal essential medium (MEM) (Invitrogen, Burlington) containing Earle's salts and L-glutamine was supplemented with 10% fetal bovine serum. The Calbiochem InhibitorSelect Protein Kinase Inhibitor Library I containing 80 receptor tyrosine kinase inhibitors and atypical kinase inhibitors was from EMD (San Diego). MP Biomedicals (Santa Ana) supplied radiolabelled ATP ([γ-^32^P]-ATP) for the *in vitro *kinase assays. HeLa 229 cells were obtained from ATCC (Manassas). *Chlamydophila pneumoniae *CWL029 and *Chlamydia trachomatis *serovar D were obtained from ATCC (cat. #VR1310 and #VR885, respectively). *E. coli *Rosetta pLysS and BL21(DE3) pLysS were from Novagen (EMD). Epidermal growth factor (EGF) and the MEK inhibitor U0126 were from Sigma (Oakville). U0126 was resuspended in DMSO immediately prior to addition to cell culture in the MEK/ERK activation experiment.

### Protein Expression and Purification

GST-PknD KD and His-FHA-2 were prepared as described [45]. Key parameters for preparing active kinase domain included cooling the *E. coli *cultures to 20°C prior to induction, inducing with 0.2 mM IPTG, and harvesting cells after 2 hours of recombinant protein expression at room temperature.

### Protein Kinase Activity

Eighty cell permeable and ATP competitive protein kinase inhibitors were purchased from EMD (San Diego). Each compound in the InhibitorSelect protein kinase library was screened at 10 μM (unless otherwise noted) in an *in vitro *PknD autophosphorylation assay. Briefly, each reaction contained 100 ng GST-PknD KD, 20 μM ATP, 5 mM MnCl_2 _and 3 μCi [γ-^32^P]-ATP in 25 mM HEPES buffer (pH 7.1) supplemented with 1× complete EDTA-free protease inhibitors, unless otherwise noted. Reactions were incubated for 90 min. at 33°C, terminated with SDS-PAGE loading buffer, separated by 10% SDS-PAGE and transferred to polyvinyldinedifluoride (PVDF) membrane. Membranes were exposed to Kodak X-OMAT film for 1-12 hours at -80°C and subsequently developed using an X-ray processor.

### ATPase Activity

ATP hydrolysis by GST-CdsN purified from glutathione-agarose beads was measured using a malachite green assay (R & D Systems). Reaction mixtures contained 100 ng of GST-CdsN, 4 mM ATP, 50 mM Tris-HCL pH 7.0, 5 mM MgCl_2_, and 10 mM KCl. Compound D7 was added to final concentrations of 1 μM, 5 μM, 10 μM and 100 μM. The reaction mixture (50 μL) was incubated at 37°C for 30 min. The reaction was stopped by the addition of 10 μL of Malachite Green Reagent A followed by 10 μL of Malachite Green Reagent B and incubated at room temperature for one minute before an OD_610 _reading was taken, according to the manufacturer's instructions.

### Immunofluorescent Microscopy and Chlamydia Growth Experiments

HeLa cells (1 × 10^5^) on coverslips in shell vials were infected with *C. pneumoniae *CWL029 (MOI of 1) using centrifugation, and replacement media containing 2 μg/mL cycloheximide was added at 1 hpi. Protein kinase inhibitors (compounds D4, D5, D6 and D7) were added to the replacement media to a final concentration of 10 μM (unless otherwise noted), for the duration of the *Chlamydia *developmental cycle (72 hours). For time course immunofluorescence (IF) experiments, compound D7 was added at 1, 15 and 24 hpi. For IF staining cell monolayers were fixed in methanol for 10 minutes at 72 hpi for *C. pneumoniae *and at 48 hpi for *C. trachomatis*. Inclusions were stained with the Pathfinder reagent, a FITC-conjugated anti-LPS monoclonal antibody (Bio-Rad, Mississauga) containing Evan's Blue counterstain. Images were captured at 400× magnification using an Olympus BX51 fluorescent microscope equipped with a color camera (Q color 5; Olympus). To determine the infectivity of *Chlamydia *grown in the presence of inhibitors, HeLa cells were infected with *C. pneumoniae *CWL029 and grown for 72 or 84 hrs in the presence of various compounds (used at 10 μM) or vehicle (DMSO 0.1%) then cells were lysed with glass beads into fresh MEM. Serial dilutions of lysates were used to infect fresh HeLa cells and inclusions were stained at 72 hr as described above.

### Salmonella Infection Assay

The effect of compound D7 on the growth of *Salmonella enterica *sv. Typhimurium SL1344 [56] in HeLa cells was determined using a cell invasion assay. Briefly, overnight bacterial cultures grown in Luria-Bertani broth (LB) were pelleted, resuspended in 1 mL PBS and diluted in DMEM containing 10% FBS to an MOI of ~1:100. An aliquot (0.5 mL) of the bacterial suspension was added to HeLa cells in a 24-well plate and incubated at 37°C in a 5% CO_2 _atmosphere for 10 minutes. The wells were then washed 3× with PBS and incubated in DMEM for an additional 20 minutes. The medium was removed and the cells were incubated in fresh DMEM containing 100 μg/mL gentamycin for 1.5 hours. Culture media was replaced with fresh DMEM containing 10 μg/mL gentamycin and either 0.1% DMSO, or 10 μM compound D4, D5, D6 or D7. At 2 and 16 hpi, intracellular bacteria were recovered by lysing HeLa cells in PBS containing 1% Triton X-100 and 0.1% SDS. Lysates were serially diluted, plated on LB plates, incubated overnight and colonies subsequently counted.

### HeLa Cell Viability

The effect of compound D7 on HeLa cell viability was determined. Briefly, 10 or 100 μM compound D7, or 0.1% DMSO, with or without cycloheximide in MEM, was added to subconfluent HeLa cells in 6-well plates. At 0, 22, 44 and 66 hours supernatants were harvested and tested for the presence of adenylyl kinase using a cytotoxicity assay (Lonza ToxiLight^® ^BioAssay, Rockland). The cytotoxicity assay was performed as per the manufacturer's protocol. Briefly, supernatants from HeLa cell cultures incubated in the presence of compound D7 or DMSO (in MEM containing cycloheximide) were tested for evidence of eukaryotic cell cytotoxicity. Aliquots (5 uL) of each supernatant were mixed with 25 uL of Adenylate Kinase Detection Reagent and samples were incubated at room temperature for 5 minutes. Relative light units (RLUs) were measured using a 20/20 n Single Tube Luminometer from Turner BioSystems (Sunnyvale). Assays were conducted in triplicate for each condition. Cell monolayers were washed with warm PBS. 0.75 mL of trypsin was added to each well, and 0.75 mL of MEM was added after complete trypsinization (trypsinization was monitored by light microscopy). Each sample was thoroughly resuspended and aliquoted into a plastic cuvette and the cell number immediately quantitated by determining the optical density at 800 nM [57] using a spectrophotomer.

### MEK/ERK Activation

To determine whether compound D7 interferes with activation of the MEK/ERK pathway, HeLa cells were exposed to compound D7, DMSO, or the specific MEK inhibitor U0126, activated with EGF and then lysates tested by Western blot for phosphorylated and total ERK as described [43]. Briefly, subconfluent HeLa cells in 6-well plates were serum-starved for 3.5 hours prior to incubation for 45 min. in either 0.1% DMSO, 10 or 100 μM compound D7 or 10 or 25 μM U0126 in serum-free MEM. Cells were then incubated with 100 ng/mL EGF in serum-free MEM for 2 minutes before being scraped in 0.5 mL ice-cold lysis buffer (50 mM HEPES pH 7.1, 150 mM NaCl, 1 mM EDTA, 1× complete EDTA-free protease inhibitors (Roche, Mississauga), 1× phosSTOP phosphatase inhibitors (Roche) and 1% Triton X-100). An equivalent amount of protein from each sample (450 ng) was separated by 10% SDS-PAGE and transferred to PVDF membrane. The membrane was blocked for 1 hour in TBS-T containing 4% BSA, and then incubated in 1:1000 anti-phospho-p44/42 MAPK (Thr202/Tyr204) antibody (#9101, Cell Signaling Technology, Danvers) overnight at 4°C in blocking buffer. The membrane was washed 3× with PBS containing 0.1% Triton X-100, incubated in 1:4000 goat anti-rabbit IgG HRP-conjugate antibody (Sigma) in blocking buffer for 1 hour at room temperature, washed and developed using enhanced chemiluminescence (ECL) reagents (Amersham, Piscataway). The PVDF membrane was then stripped of antibody, blocked, re-probed with 1:1000 anti-p44/42 MAPK antibody (#9102, Cell Signaling Technology) and developed as above.

### Transmission Electron Microscopy

HeLa cells (1 × 10^6^) in 9 cm^2 ^wells of six-well plates were infected with *C. pneumoniae *CWL029 at a multiplicity of infection of 1. Compounds were added at 1 hpi and cells harvested at 48 hpi. Cells were fixed overnight at 4°C in 0.1 M sodium cacodylate buffer containing 2% gluteraldehyde, embedded in araldite resin and thin sections were viewed using a Jeol JEM 1200EX electron microscope at 12,000× magnification.

## Abbreviations

EB: elementary body; ECL: enhanced chemiluminescence; hpi: hours post infection; IB: intermediate body; IF: immunofluorescence; LB: Luria-Burtani broth; PB: persistent body; PknD: protein kinase D; RB: reticulate body; T3SS: type III secretion system.

## Authors' contributions

CBS conducted the ATPase assay and DCB conducted the HeLa cell cytotoxicity analysis and prepared the associated bar graph. BKC conducted the *Salmonella *growth experiment and prepared the associated bar graph. JBM contributed to study conception and design and drafting the manuscript. DLJ contributed to study conception and design, carried out all other experiments and drafted the manuscript. All authors read and approved the final manuscript.
